# Current challenges and improvements in assessing the immunogenicity of bacterial vaccines

**DOI:** 10.3389/fmicb.2024.1404637

**Published:** 2024-07-09

**Authors:** Giulia Fantoni, Giuseppe Boccadifuoco, Federica Verdirosa, Eleonora Molesti, Alessandro Manenti, Emanuele Montomoli

**Affiliations:** ^1^VisMederi S.r.l., Siena, Italy; ^2^Department of Biotechnology, Chemistry and Pharmacy, University of Siena, Siena, Italy; ^3^Department of Molecular and Developmental Medicine, University of Siena, Siena, Italy

**Keywords:** serum bactericidal assay, luminescent SBA, opsonophagocytosis assay, vaccine, serological assay

## Abstract

The increase in antimicrobial-resistant bacterial strains has highlighted the need for a new vaccine strategy. The primary goal of a candidate vaccine is to prevent disease, by inducing a persistent immunologic memory, through the activation of pathogen-specific immune response. Antibody titer is the main parameter used to assess the immunogenicity of bacterial vaccine candidates and it is the most widely used as a correlate of protection. On the other hand, the antibody titer alone cannot provide complete information on all the activity mediated by antibodies which can only be assessed by functional assays, like the serum bactericidal assay and the opsonophagocytosis assay. However, due to the involvement of many biological factors, these assays are difficult to standardize. Some improvements have been achieved in recent years, but further optimizations are needed to minimize inter- and intra-laboratories variability and to allow the applicability of these functional assays for the vaccine immunogenicity assessment on a larger scale.

## 1 Introduction

After the emergency of the COVID-19 pandemic, the focus on infectious diseases is shifting to another global public health threat caused by the rise of antimicrobial-resistant (AMR) bacterial strains. At the beginning of the 20th century, bacterial diseases were the highest cause of mortality and morbidity. The 1940–1980 period was characterized by a continuous discovery of new antibiotics, the so-called “miracle drugs,” which increased life expectancy and was perceived by society as a milestone achievement in microbiological research (Baquero, 2021). From the mid-1980s, the focus of the pharmaceutical industry shifted to other drug development, and the discovery of new antibiotics was remarkably reduced. Therefore, since the emergence of antibiotic resistance was no longer compensated by new anti-microbial drug discoveries, bacteria became again a global threat to modern health care, raising concerns of national and international public health organizations, including the World Health Organization (WHO) (Baquero, 2021). For this reason, during the last few years, vaccines have become a valuable alternative to prevent pandemics caused by AMR bacteria, for which currently available treatments are no longer or less effective. Vaccines can mitigate directly and indirectly the antimicrobial resistance consequences. Vaccines can prevent diseases by the elicitation of the immune response, reducing antimicrobial drug consumption, and the development of resistance. This is particularly important considering community and healthcare-associated infections with multidrug-resistant pathogens. Additionally, the use of viral vaccines reduces the need for antibiotic treatments, that may occur in case of secondary bacterial infections (Rosini et al., 2020). However, bacteria are much more complex organisms than viruses and represent a more difficult target for vaccine development, today only a limited number of vaccines are licensed and applied against bacterial infections. Despite these difficulties, the WHO encourages the development of new therapies and vaccines targeting AMR bacteria strains (Tacconelli et al., 2018). However, for bacterial vaccine advancement, appropriate assays for immunogenicity assessment are needed. Progress in antigen discovery must go along with the set-up of robust and reliable methods of evaluation performed during the strict approval process.

## 2 Which aspects are investigated to evaluate vaccine immunogenicity?

**Figure 1:**
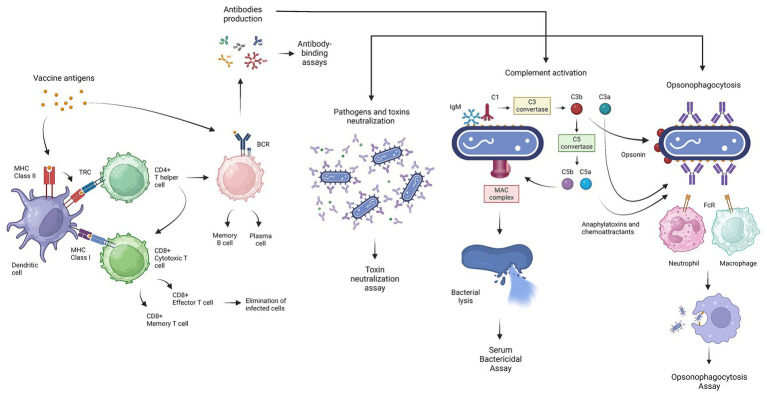
Graphical representation of the generation of the basic immune response induced by vaccination, the main functions mediated by antibodies against bacterial infection, and the related serological test. Antigen presentation, followed by vaccine administration, induces the selection of antigen-specific B- and T-cells. The dendritic cells (DCs) process the antigens and present them via MHC class I or MHC class II, promoting differentiation into cytotoxic T-cells (CD8+) and helper T cells (CD4+), respectively. Cytotoxic T-cells (CTLs) mediate the lysis of autologous infected cells, while helper T-cells stimulate other immune cells, like the B-cells, through cytokine secretion. B-cells can directly interact with the vaccine antigens and produce antibodies, which mediate several functions necessary to overcome bacterial infections. The main functionality of antibodies includes neutralization of pathogens and toxins, complement activation which leads to bacterial lysis through the formation of the membrane attack complex (MAC), and, finally, opsonophagocytosis mediating the elimination of bacteria by phagocytic cells. Antibody level is estimated through antibody-binding assays (ELISA), while functionality is assessed using the toxin neutralization test, the serum bactericidal assay (SBA), and the opsonophagocytosis assay (OPA). This figure was created using BioRender.com.

Vaccination aims to induce an immune response able to control exposure to an infectious agent and clear or prevent the infection. Ideally, a candidate vaccine should induce the adaptive immune response, represented by both humoral and cell-mediated immunity. The antigen presentation followed by vaccine administration, induces the selection of antigen-specific B-and T-cells in separate ways (Figure 1) (Kapingidza et al., 2020). The B-cell receptor (BCR) allows direct interaction with the antigen, without T-cell involvement. The humoral immunity is based on the capacity of the B-cells to secrete antibodies. Antibodies have two functional domains, called the antigen-binding fragment F(ab’)2 and the crystallizable fragment (Fc) which are responsible for the specificity and functionality, respectively. The basic function of the F(ab’)2 fragment is the binding of antigenic epitopes via complementarity-determining regions (CDRs) (Mix et al., 2006). The Fc domain passes through structural variations identified as five isotypes (IgM, IgD, IgG, IgA, and IgE) which are responsible for distinct innate immune effector functions (Kapingidza et al., 2020). Antibodies variety is generated during Ag-dependent B-cell maturation in peripheral lymphatic tissues by isotype class switching and somatic hypermutation (Mix et al., 2006). The Fc fragment mediates several antibody functions, such as receptor-mediated phagocytosis, cytotoxicity, the release of inflammatory mediators, the transport through the mucosa, and complement activation (Figure 1) (Mix et al., 2006; Jiskoot et al., 2019). It follows that a strong antibody–antigen affinity and specificity may generate greater potency and efficacy against bacterial infections (Goulet and Atkins, 2020). The antibody-mediated neutralization represents one of the simplest mechanisms involved in protection against bacteria. The F(ab’)2 domains bind the specific pathogen antigen, inhibiting the host-cell invasion or bacterial toxin effect. Moreover, antibodies hold back pathogen pathogenesis, with the inhibition of microbial virulence factors like biofilm formation (Lu et al., 2018).

The complement activation represents one of the most crucial functions mediated by antibodies. The complement system consists of a complex of almost 50 plasma and surface proteins and is part of the innate immune system which cooperates with antibodies for the rapid elimination of invading bacteria. In general, the complement activation induces three different enzymatic cascades called, the classical, mannan-binding lectin (MBL) and alternative pathways. The classical complement pathway is triggered by the antibody’s interaction with the bacterial surface antigens. The C1 complex interacts with the Fc region of pentameric IgM or IgG complex and leads to the formation of the C3 convertase enzyme which transforms C3 into C3a and C3b. A high concentration of C3b switches the substrate from C3 convertase to C5 convertase, which catalyzes the cleavage and the release of C5a and C5b. The association of the C5b with C6, C7, and C8 allows the binding and then the polymerization of C9, generating the membrane attack complex (MAC) (Heesterbeek et al., 2018). The MAC is a ring-structured pore that can directly kill Gram-negative bacteria without the involvement of immune cells through osmolysis (Xie et al., 2020). The gram-positive bacteria are less susceptible to this mechanism for the presence of the peptidoglycan outer layer. In this case, the killing action is led by the strong chemoattractant C5a which recruits phagocytes in the infection site, and by the C3b opsonized particles that enhance the phagocytosis. The C3-derived products are also responsible for a long-term immune response, stimulating the adaptive immune response through the antigen presentation to B-cells and antigen-presenting cells (APC) placed in the lymphoid organs (Heesterbeek et al., 2018; Lu et al., 2018). Then, the MBL pathway is triggered by surface-specific recognition of molecules like collectins and ficolins uniquely present in bacteria and fungi. These molecules in complex with the MBL-associated serine proteases (MASPs) lead to the formation of C3 convertase. Lastly, in the alternative pathway, the factor B and D proteins also play a role in the amplification loop of C3b molecules. It is also considered that in the third recognition path, factors B and D can react with spontaneous hydrolyzed C3 (C3_H2O_) to obtain C3 convertase (Heesterbeek et al., 2018; Lu et al., 2018). In general, all three pathways lead to the formation of C3 and C5 convertase which catalyze the formation of the C3a and C5a. The C3a and C5a are anaphylatoxins and leukocyte chemoattractants, that bind their specific receptor (C3aR, C5aR1, and C5aR2) on immune cells to stimulate and modulate the inflammatory response. Although anaphylatoxins play an important role in protection against certain infections, dysregulation has been associated with several diseases related to inflammatory disorders, like allergy, autoimmunity, neurodegenerative diseases, and cancer (Nandakumar et al., 2010; Ghosh and Rana, 2023). Consequently, the C5a and C3a elicitation can be taken into consideration to evaluate the possibility of allergic reactions and hypersensitivity, during vaccine immunogenicity evaluation (Nicaise-Roland et al., 2022).

Concerning the ability of the immune response to face bacterial infections, cell-mediated immunity also plays an important role. The T-cells are unable to interact with antigens directly, but depend on the APC, like dendritic cells (DC) or macrophages, to process antigens and present through the MHC class I or MHC class II, enhancing the differentiation to cytotoxic T-cells (CD8+) and T helper cells (CD4+), respectively (Salerno-Gonçalves and Sztein, 2006). Cytotoxic T-cells (CTLs) mediate the lysis of autologous cells infected by intracellular pathogens, and T-helper cells stimulate other immune cells through the secretion of cytokines and by cell-to-cell interactions. Based on the profile of the secreted cytokines, the T-cells are classified as T helper 1 (Th_1_) or T helper 2 (Th_2_) (Jiskoot et al., 2019). Th_1_ response stimulates the cellular-based immune response, while Th_2_ response leads to the humoral immune response. The Th_1_-cells produce cytokines, such as interferon-gamma (IFN-γ) and tumor necrosis factor alfa (TNF-α), which potentiate the effector function of phagocytes and increase inflammation (Jiskoot et al., 2019). On the other hand, Th_2_ cells developed under the influence of IL-4 signaling, produce another set of cytokines that support B-cells proliferation and differentiation (Jiskoot et al., 2019). For this reason, Th_2_ cells have been associated with increased humoral responses. However, the cytokines produced by Th_2_ cells have been linked to IgE production; consequently, the elicitation of memory Th_2_ cells has become an important focus in the design of novel candidate vaccines, to evaluate and mitigate allergic reactions (Jiskoot et al., 2019). Therefore, a vaccine candidate that has a balanced Th_1_/Th_2_ response can be considered optimal (Salerno-Gonçalves and Sztein, 2006). However, current advances in the understanding of intracellular bacterial infection revealed that the immune response is more complex than the Th1/Th2 dichotomy and recognizes the role of T-helper 17 cells (Th17) in antimicrobial host defense. The Th17 is a subset of pro-inflammatory T-helper cells defined by their production of interleukin 17 (IL-17). IL-17 exhibits proinflammatory effects and is known to mediate the immunomodulation of DC, recruitment of neutrophils, and promoting the CTL and Th1 response, which is crucial against intracellular bacteria (Li et al., 2018). Therefore, the Th17/IL-17 elicitation represents an important parameter to evaluate vaccines for intracellular bacteria. The Natural Killer (NK) cells are another separate lymphocyte lineage that shows both cytotoxicity and cytokine-producing effector roles. A further function of the antibodies is the enhancement of the cytotoxic destruction of the bacterial cells through the so-called antibody-dependent cellular cytotoxicity (ADCC), mediated by NK cells. The NK cells, activated by the binding with the Fc portion of the antibody, release perforin and granzyme which lead to the death of infected or tumor cells (Vivier et al., 2008). The elicitation of efficient T-cells and the generation of memory T-cells represent another challenge in vaccine development. Indeed, modern vaccines may not efficiently elicit long-term T-cell immunity due to the short persistence of the antigens. To improve this aspect, DNA-based vaccines, viral vectors, prime-boost regimes, and adjuvant combinations, have been proposed (Corrado and Pearce, 2022). In this context, the most used techniques for the evaluation of the cell-mediated immunity response are the Enzyme-Linked ImmunoSpot assay (ELISpot) and flow cytometry applied to intracellular cytokine staining (ICS) (Salerno-Gonçalves and Sztein, 2006).

The last important function triggered by antibodies is the enhancement of opsonophagocytosis. These antibody-coated pathogens are labeled for phagocytosis by neutrophils and macrophages (Figure 1). In the case of antibody-dependent cellular phagocytosis, the Fc receptor recognizes the antibodies binding the bacterial surface and starts intracellular signaling which leads to the actin cytoskeleton rearrangement and the formation of the phagosome (Pidwill et al., 2021). Moreover, the Fc receptor recognition activates signaling of the immunoreceptor tyrosine-based activation motifs (ITAMs), which lead to the antigen presentation to the T-cells, and in some cases activates the immunoreceptor tyrosine-based inhibition motifs (ITIMs) for the retention of the whole pathogen antigens to be transferred to B-cells and induce also a humoral immunity (Lu et al., 2018).

## 3 Available bacterial vaccines

The primary goal of a candidate vaccine is to prevent disease by inducing a persistent immunologic memory through the activation of pathogen-specific immune response. In particular, long-term protection involves memory cell activation that rapidly triggers a secondary immune response. Memory B-cells are responsible for the secretion of high-affinity functional antibodies, while memory T-cells are capable of rapid expansion and cytotoxic properties (Corrado and Pearce, 2022). Vaccine boosters aim to increase the quality, quantity, and persistence of the immune response inducing a secondary response (Montero et al., 2024). However, this depends on the vaccine formulation and the target pathogen. The presence of adjuvants in the formulation can induce an early activation of innate immunity which then turns into higher antibody and cellular responses to the vaccine antigens (Del Giudice et al., 2018). In general, adjuvants are useful for those vaccines formulated with purer components, like purified recombinant antigens, which represent a safer option but also show lower immunogenicity, in contrast to other types of vaccines (Del Giudice et al., 2018). In the case of the hepatitis B vaccine, the presence of adjuvant systems has been associated with positive regulation of genes associated with IFN-γ-related responses and the innate cell compartment (De Mot et al., 2020). The complexity of bacteria makes them more challenging targets for vaccine development compared to viruses (Osterloh, 2022). However, they naturally contain various immunostimulatory components that can be exploited as in-built adjuvanticity (Del Giudice et al., 2018).

Currently, five main groups of bacterial vaccines are available: whole-cell antigen (WCA), polysaccharide/protein conjugates; recombinant proteins including toxoids; live attenuated vaccines (LAV); and, more recently introduced, bacterial outer membrane vesicles (OMVs) (Table 1) (Jiskoot et al., 2019). The WCA vaccine is based on the administration of inactivated bacteria (heating, irradiation, or chemical) and predominantly induces the activation of the B-cells assisted by T-helper cells for the antibodies production. For this reason, WCA immunization is efficiently used for vaccination against extracellular bacteria, like *Vibrio cholerae* (Dukoral and Shanchol) (Song et al., 2021; Osterloh, 2022). However, antibodies may lack effectiveness against intracellular pathogens, due to the difficulties in reaching the target. An efficient defense against intracellular bacteria requires the activation of cytotoxic CD8+ T-cells, mediated by the MHC class I pathway, and Th1/Th17 response. The polysaccharide conjugate vaccines are based on bacterial capsular polysaccharides conjugated with a carrier protein. The polysaccharides alone cannot be processed and displayed on MHC molecules like proteins, consequently, it activates an immune response T-cell independent. The polysaccharide conjugation with a protein carrier enhances the engagement of CD4+ T-helper cells through the interaction with the MHC class II of B-cells which leads to higher affinity and class-switched antibodies (Rappuoli, 2018). Examples of bacterial polysaccharide conjugate vaccines are vaccines against *H. influenzae* type b (PedvaxHIB, ActHIB, HibTITER, proHIBiT), pneumococci (Prevnar (PVC7), Pneumovax 23(PPV)), meningococci (Menactra, Menveo, MenQuadfi) (Thisyakorn et al., 2014; Mbaeyi et al., 2020; Slack et al., 2020). The toxoid vaccines (DTaP) are composed of inactivated exotoxins released by bacteria like *Corynebacterium diphtheriae, Clostridium tetani, Bordetella pertussis*, induce the activation of B cells and antibody production with the cooperation of T-helper cells (Domenech De Cellès et al., 2019; Osterloh, 2022). Differently, the LAVs are based on microorganisms that have lost pathogenicity but maintain the capacity for transient intracellular replication, they can activate the cytotoxic CD8+ T-cells through the interaction with MHC class I of infected cells, reaching an efficient defense against intracellular bacteria (Osterloh, 2022). Examples of such attenuated vaccines are *Mycobacterium tuberculosis* (BCG), *Salmonella typhi* (Vivotif), and *Vibrio cholerae* (Vaxchora) (Fraser et al., 2007; Roy et al., 2014; Mosley et al., 2017). The OMVs carry many bacterial antigens preserving the features of the bacterial membrane, that when transferred into the cytosol of target cells have the potential to elicit T-cell responses, including cytotoxic CD8+ T-cell responses (Osterloh, 2022). The four licensed vaccines based on OMVs are all directed against *N. meningitidis* serogroup B bacteria (Bexsero/4CMenB, VA- MENGOC-BC, MenBVac, MeNZB) (Castilla et al., 2023).

**Table 1:** Five main types of available bacterial vaccines with related induced immunity response (Osterloh, 2022), an example of available vaccines, assay for immunogenicity evaluation and efficacy.

Type of bacterial vaccines	Induced immune response	Bacteria and example of licensed vaccine	Assay for immunogenicity evaluation	Efficacy	Ref.
Whole cell antigen (WCA)	WCA can be obtained by heating, irradiation, or chemical inactivation of the bacteria, such as the treatment with formaldehyde or alkylating reagents. WCA immunization induces the activation of B cells and antibody production as well as the activation of CD4^+^ T cells. Efficient for vaccination against extracellular bacterial infection.	***Vibrio cholerae*** Dukoral: a mixture of killed *V. cholerae* O1 bacteria with the recombinant B-subunit of cholera toxin (CTB). Shanchol: comprises four strains, one of *V. cholerae* O139 (4260B) and three of *V. cholerae* O1.	ELISA and SBA (Vibriocidal Titer).	At the six-month interval, the estimated efficacy for WC-BS was 85% (95% CI: 62–94, *p* < 0.0001) and consistent across age groups However, the cumulative efficacy for WC-BS dropped to 62% (lower bound 47%, *p* < 0.0001) at the one-year interval, and 50% at the completion of the three-year follow-up. The cumulative protective efficacy of Shanchol at five years is 65% (95% CI: 52–74, *p* < 0.0001).	Song et al. (2021)
Polysaccharide / protein conjugates	The polysaccharide conjugate vaccines are based on bacterial capsular polysaccharides conjugated with a carrier protein. The polysaccharides alone cannot be processed and displayed on MHC molecules like proteins, consequently, it activates an immune response T-cell independent. The polysaccharide conjugation with a protein carrier enhances the engagement of CD4+ T-helper cells through the interaction with the MHC class II of B-cells which leads to higher affinity and class-switched antibodies. Efficient for vaccination against extracellular bacterial infection.	***Haemophilus influenzae* type b (Hib)** Monovalent vaccines: PedvaxHIB: *Neisseria meningitidis* outer membrane protein complex (Hib-OMP). ActHIB: tetanus toxoid (Hib-TT). HibTITER: the nontoxic mutant of *Corynebacterium diphtheriae* toxin (Hib-CRM197). proHIBiT: diphtheria toxoid (Hib-DT).	ELISA and SBA	All 4 vaccines were shown to be efficacious against invasive Hib disease in clinical trials, with vaccine efficacy varying between 87 and 100% for Hib-DT, 90 and 100% for Hib-CRM, 93 and 95% for Hib-OMP, and 93 and 100% for Hib-TT after at least 2 doses.	Slack et al. (2020)
		***Neisseria meningitidis*** Menactra: MenACWY-D polysaccharide diphtheria toxoid conjugate vaccine. Menveo: MenACWY-CRM oligosaccharide diphtheria CRM197 conjugate vaccine. MenQuadfi*: MenACWY-TT* polysaccharide tetanus toxoid conjugate vaccine.	SBA	Among infants who received two doses 89–96% achieved an hSBA titer ≥1:8 against serogroup A, ≥98% against serogroup C, 81–92% against serogroup W, and 95–97% against serogroup Y. Among infants the proportions of infants with an hSBA titer ≥1:8 was 89–96% for serogroup A and ≥ 95% for serogroups C, W, and Y after the fourth dose. Among children who received a single dose at age 2–9 years, the proportions who achieved hSBA titers ≥1:8 1 month after vaccination were 86% for serogroup A, 98% for serogroup C, 95% for serogroup W, and 99% for serogroup Y.	Mbaeyi et al. (2020)
		***Streptococcus pneumoniae*** Prevnar (PCV7): contains CRM197 diphtheria toxin-conjugated polysaccharides from serotypes 4, 6B, 9 V, 14, 18C, 19F, and 23F. Pneumovax (PPV23): Pneumo23^®^ (PPV23; Sanofi Pasteur) is a pneumococcal vaccine containing unconjugated polysaccharide from 23 serotypes (1, 2, 3, 4, 5, 6B, 7F, 8, 9 N, 9 V, 10A, 11A, 12F, 14, 15B, 17F, 18C, 19A, 19F, 20, 22F, 23F, and 33F).	ELISA and OPA.	The boost with PPV23 is immunogenic and well tolerated in healthy toddlers primed with PCV7.	Thisyakorn et al. (2014)
Toxoid and recombinant proteins	Toxoids are composed of inactivated exotoxins released by bacteria. Immunization induces the activation of B cells and Th cells for antibody production. Efficient for vaccination against extracellular bacteria.	***Corynebacterium diphtheriae, Clostridium tetani, Bordetella pertussis*** DTaP: Diphtheria and tetanus toxoids (inactivated toxins) and acellular pertussis antigens.	ELISA, Toxin-neutralization assay	Effectiveness in children aged 5 to 9 years exceeds approximately 75%, and more than 65% of children remain immune to pertussis 5 years after the last dose of DTaP.	Domenech De Cellès et al. (2019)
Live attenuated bacteria (LAV)	LAVs are microorganisms that have lost pathogenicity but still have the capacity for transient intracellular replication where bacterial antigens are more likely accessible for the MHC-I presentation pathway and the induction of CD8^+^ T cell responses. Efficient for vaccination against intracellular pathogens.	***Mycobacterium tuberculosis*** BCG: *M. bovis* attenuated strain.	ELISpot, Intracellular cytokine staining	A meta-analysis of observational studies of primary BCG vaccination showed a pooled estimate of 27% efficacy against initial *M. tuberculosis* infection and 71% efficacy against tuberculosis disease.	Roy et al. (2014)
		***Salmonella typhi*** Vivotif: Ty21a attenuated strain.	ELISA	The cumulative efficacy at 3 years of 51%.	Fraser et al. (2007)
		***Vibrio cholerae*** Vaxchora: attenuated cholera bacterium serogroup O1.	ELISA and SBA (Vibriocidal Titer).	90.3% effective against *V. cholerae* O1 10 days after vaccination and 79.5% effective 90 days after vaccination.	Mosley et al. (2017)
Outer membrane vesicles (OMVs)	They transport antigens that have the potential to elicit T-cell responses, including CTL cell responses. When the OMVs are transferred into the cytosol of target cells, carrying pathogen-associated molecular patterns (PAMPs) that can stimulate innate immune responses and induce CTL cell responses capable of fighting intracellular bacteria.	***Neisseria meningitidis* Serogroup B** Bexsero (4CMenB): OMV derived from a MenB outbreak strain (B:4: P1.7–2.4) with factor-H-binding protein, neisserial heparin-binding antigen, and neisserial adhesin A.	SBA, MATS, MEASURE	Complete vaccination with 4CMenB was 76% effective in preventing meningococcal disease caused by any serogroup, 71% effective in preventing disease caused by serogroup B, and 92% effective in preventing disease caused by non–serogroup B meningococci.	Castilla et al. (2023)

As a consequence of emerging AMR bacteria, the development of novel vaccine candidates against these bacterial targets is strongly encouraged by the public health authorities and a list of bacterial priority pathogens is available and updated by WHO. Vaccine strategy requires the inclusion of multiple serotypes, different adjuvants, and the continuous discovery of immunogenic epitopes. Bacteria possess a variety of antigens whose immunogenic potential is often unknown, and it is unclear which antigen can elicit a protective and long-lasting immune response (Poolman, 2020). There are several limitations to new bacterial vaccine development: the lack of a known correlate or surrogate of protection, the need for appropriate animal models, and the improvement in certain vaccine formulations that can increase the efficacy in high-risk groups such as infants and elders. Furthermore, intracellular bacteria represent a more challenging target for vaccine development, as the protection requires the activation of the cell-mediated immune response, besides the antibody production (North, 2020). Vaccine development is a process that requires multiple steps and needs to take into consideration multiple aspects besides the antigen discovery. The first aspect is the epidemiological impact determined by the disease, the existence of alternatives to treat the disease, and the cost/benefit associated. Fundamental is the involvement of research groups and institutions that can be aware of the stage of the scientific knowledge of the selected disease and can identify gaps in vaccine development. Lastly, significant is the existence of laboratories capable of carrying out the process from pre-clinical to phase III clinical studies in compliance with Good Clinical Practice (GCP) and others that meet Good Manufacturing Practice (GMP) for the production of the vaccines. After considering all these aspects, on average it takes 10 to 15 years to introduce a new vaccine to the market. The development of a vaccine is pyramidal, for every success there are many failures, where most failures occur in the preclinical and phase I stages of clinical studies (Pollard and Bijker, 2021). Traditional strategies for bacterial vaccine development aimed to induce a strong humoral response, prioritizing the use of antibody-binding assays to evaluate immunogenicity. However, binding assays, like ELISA, can only measure the level of antibodies in the serum of the patient, which may not necessarily reflect the degree of protection. Likely, the failure of some bacterial vaccines led to a broader view including the evaluation of multiple aspects of the immune response. For anti-viral vaccines, immunogenicity can be assessed through non-functional and functional assays (Trombetta et al., 2014). For example, the neutralization assay has been applied to evaluate neutralizing antibodies to SARS-CoV-2. The fast approval of the COVID-19 vaccines was partly related to the reliability of these serological assays (Manenti et al., 2022). In this case, the availability of well-established functional assays, recognized by regulatory agencies, made the immunogenicity evaluation more straightforward. However, in the case of bacterial vaccines, functional assays involve critical biological components, such as live bacteria and differentiated immune cells, which are more difficult to standardize in comparison to the antibody-binding counterparts (Feavers and Walker, 2010). Further advancement in functional assays that evaluate the bactericidal and opsonization ability of the antibodies could help the development and approval of new candidate vaccines.

## 4 Immunologic correlates of vaccine-induced protection against bacteria

Immunogenicity is assessed at all clinical stages of the vaccine approval journey. Considering the complexity of the immune response, it is difficult to determine what are the specific immunological factors triggered against a specific pathogen. The correlate of protection (CoP) is defined as a laboratory marker of immune response, that correlates with the protection from infection, disease, or other defined endpoints (Plotkin, 2008). Considering the redundancy of the immune system, more than one vaccine-induced response can be a CoP (Plotkin, 2020). The establishment of a correlate of protection is fundamental for vaccine development strategies, as it should meet certain criteria to be accepted by regulatory agencies. The absolute CoP is a defined threshold considered for vaccine efficacy, while the relative CoP is an indicative level where the efficacy may occasionally fail. When more than one immune function is involved in protection is defined as co-correlate (Plotkin, 2020). In general, the majority of vaccines induce protection through a synergic action of antibodies and cellular immunity, which may prevent the pathogen’s diffusion through multiple mechanisms. The *in-vitro* evaluation of functional antibodies is the key to better characterize the features of the elicited humoral responses against bacterial pathogens (Boero et al., 2023). Serological assays able to measure the antibody’s functionality may be critical for vaccine licensure, however, there is the need for further optimizations and standardization to overcome the key barriers to the discovery of pathogen-specific CoP. Consequently, the principal CoP for bacterial infection is still represented by the antibodies measured by ELISA and there is not a defined threshold for protection for many diseases (Ipsen, 1946; Herrera et al., 2022). Usually, the toxin neutralization assay is employed to predict the protection against toxin-producing bacteria. However, in the case of anthrax bacillus and pertussis, a better correlation can occur with the assessment of specific IgG antibody levels against the toxins. Protection against pertussis also involves the cellular response elicitation (Plotkin, 2020). CoPs for enteric vaccines include antibody responses but also undefined responses in the intestine (Holmgren et al., 2017). In the case of *Shigella* spp., protection involves bactericidal and opsonophagocytic response and intestinal IgG and IgA antibodies against the O antigen (Holmgren et al., 2017). For pneumococcal conjugate vaccines have been established a protective cut-off value of IgG antibodies for all serotypes but protection involve also the antibodies’ opsonophagocytic ability evaluated through opsonophagocytosis assay (OPA) (Oosterhuis-Kafeja et al., 2007). For meningococcal vaccines, clinical efficacy trials are not feasible due to the low incidence of Invasive Meningococcal Disease (IMD). In 1969, Goldschneider et al. reported that the presence of serum bactericidal antibodies is predictive of protection from the disease and today the serum bactericidal assay (SBA) is considered the surrogate of protection (Goldschneider and Artenstein, n.d.). However, functional assays are more complex than antibody-binding assays; they require multiple biological factors that are not well or easy to standardize. It is also commonly agreed that traditional SBA and OPA assays can be labor-intensive, time-consuming, and require specialized equipment and well-trained personnel. Moreover, the need for international standards is a limiting factor for functional test qualification and validation restricting their employment for vaccine efficacy assessment (Toh et al., 2021).

## 5 Current serological assays used for the evaluation of bacterial vaccine immunogenicity

The evaluation of immunological response is a critical aspect of vaccine development, and it is required by all regulatory agencies for vaccine licensure. In the case of bacterial vaccines, the immunogenicity is evaluated through antibody-binding assay, SBA, and OPA assays which have been discussed more in detail in the sections below.

### 5.1 Antibody-binding assay

In the case of bacterial vaccines, the antibody titer is the major parameter evaluated for assessing the immunogenicity (Osterloh, 2022). The Enzyme-Linked Immunosorbent assay (ELISA) represents the most widely serological assay used for the quantification and identification of specific antibody responses. Antibody concentration, evaluated by ELISA assay, is considered a reliable correlate of protection for most bacterial vaccines (Plotkin, 2020). Similarly, the flow cytometry tool can be used to assess antibody binding to whole pathogens rather than to specific antigens. In addition, the multiplex technologies can allow the simultaneous identification and quantification of the antibody’s binding to a substantial number of antigens related to several bacteria serotypes. For example, the Luminex platform takes advantage of the xMAP^®^ microsphere technology, where beads are internally dyed with two or three spectrally distinct fluorochromes and covalently coupled with different capture molecules (Dunbar, 2023). These specific combinations allow distinct analysis of multiplex data and the simultaneous assessment of the antibody responses to multiple antigens. This is particularly useful in the case of polyvalent vaccines like the Pneumococcal Conjugate Vaccine (PCV), which has broad coverage and individual serological immunoassay would be extremely time-consuming and laborious (Dunbar, 2023). However, concerning PVC vaccines, the ELISA remains the gold standard for the detection of capsule-specific IgG antibodies (LaFon and Nahm, 2018). Although, antibodies against pneumococcal polysaccharide are the primary mediators of bacterial opsonization and killing, other antibodies lacking functional activity are present in the serum. Indeed, first-generation ELISA tended to overestimate antibody levels due to the presence of non-functional antibodies. At the same time, for third-generation ELISA the specificity has been improved via pre-absorption with both pneumococcal cell wall and 22F polysaccharide to remove non-protective antibodies (Wernette et al., 2003; LaFon and Nahm, 2018). Despite the improvements, it occurred that IgG antibodies did not reflect the level of protective immunity and OPA data confirmed that these antibodies were non-functional. For this reason, toxin neutralization assay, SBA and OPA, can provide information on the functional activity of the antibodies against the bacteria (Figure 1) (Feavers and Walker, 2010).

### 5.2 The serum bactericidal assay

**Figure 2:**
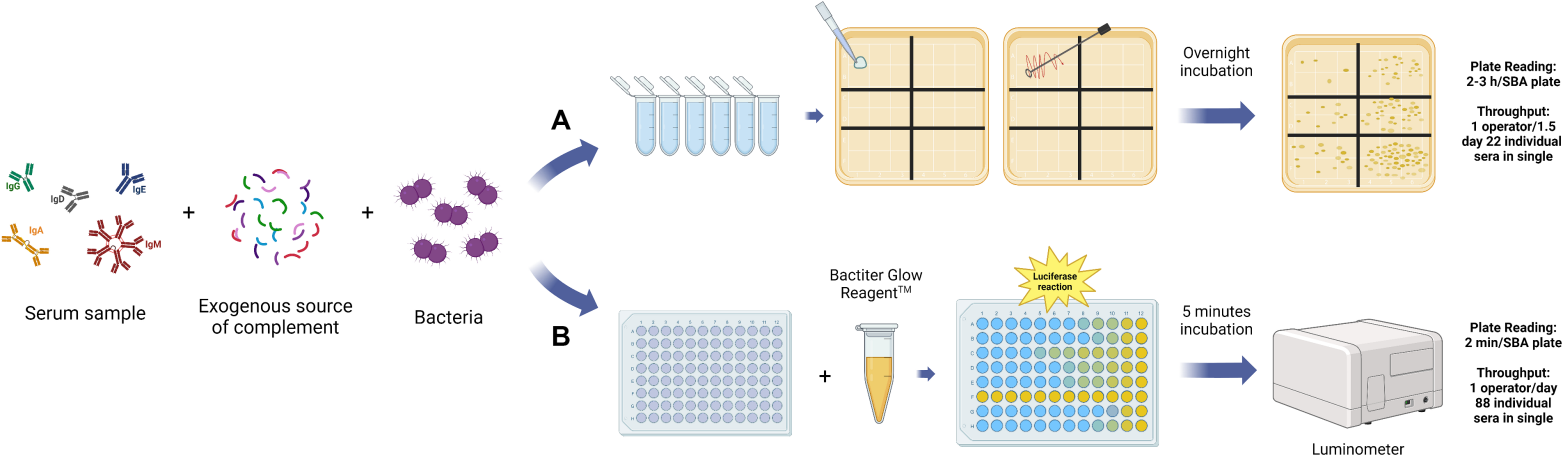
Graphical representation of the main steps for the Conventional SBA **(A)** and Luminescence-based SBA **(B)**. The assay reaction mix is the same for the two versions of the SBA assay, composed of the serially diluted serum samples from the immunized patients, an exogenous source of complement, and bacteria. After the incubation, in the case of the C-SBA **(A)**, the reaction mix of each well is plated on agar and incubated overnight. The day after the colony counting of each plate is performed. The readout is the colony forming unit (CFU) value for each of the sera dilution points, which is proportional to the number of surviving bacteria. In the case of L-SBA **(B)**, the BacTiter GlowTM reagent is added to the assay plate, after the incubation, and the luminescence value is recorded within 5 min using a luminometer. The read-out is the luminescence value for each sera dilution point, which is proportional to the number of survival bacteria. The L-SBA is a high throughput method that allows the test of 88 sera samples in single per operator in 1 day, in contrast to the C-SBA with only 22 sera samples in single per operator in 1,5 days (Aruta et al., 2021). This figure was created using BioRender.com.

The Serum Bactericidal Assay (SBA) aims to measure the capability of pathogen-specific antibodies present in the serum to activate the complement classical pathway, trigger the MAC formation, and consequently kill the target organism (Feavers and Walker, 2010). In general, the assay involves live bacteria, usually grown until the mid-exponential phase, incubated with serially diluted sera, and an exogenous source of complement (Figure 2) (Feavers and Walker, 2010). The complement, bacteria, and their interaction, are the main biological factors difficult to standardize. On the other hand, the SBA makes it possible to evaluate the antibody’s capacity to effectively neutralize the bacteria present in the reaction. This is a significant advantage compared to the use of binding-assays which can only estimate the specific antibodies concentration but not their functional capacity. In the classical or conventional version of the SBA (C-SBA), after the incubation time, the reaction mix is plated in solid agar plates and incubated overnight (Figure 2A). In this case, bacterial survival is evaluated through the count of the Colony Forming Units (CFU). The readout is expressed as the reciprocal titer of the serum which kills ≥50% of the bacteria when compared to the negative control (Borrow et al., 2020). Bacteria can be naturally susceptible or resistant to complement-mediated killing, even in the absence of antibodies. If the bacteria are too sensitive or resistant to complement action, the SBA assay is not applicable (Toh et al., 2021). Theoretically, the complement source of choice should be from the same host species, as the assay should mimic the real immune response during the infection (Feavers and Walker, 2010). For this reason, the SBA assay used to evaluate the bactericidal activity against human host pathogens should involve human serum as a complement source. However, in the case of *Neisseria meningitidis,* the human complement from healthy donors can have intrinsic bactericidal activity against meningococci and the procurement of large amounts of those lots with normal hemolytic activity represents a problem (Borrow et al., 2020; Findlow et al., 2022). Consequently, the human complement source must be validated in advance for each isolate to avoid interference with the assay result. The potential solution is the use of a complement source different from the host species. The rabbit complement is the most widely used as it was found to be a reliable and suitable source of complement for SBA (rSBA). However, while the rSBA has been used for the evaluation of MenACWY vaccines, in the case of the MenB vaccine only human complement (hSBA) is appropriate due to the higher SBA titer obtained with rabbit complement (Plotkin, 2008). In the case of encapsulated bacteria, like *Haemophilus influenzae type b* (Hib) and *N. meningitidis* type A and C (men A, C), the SBA is considered the co-CoP together with the ELISA assay (Hib: ELISA Long term: ≥ 1.0 g/mL, Short term: ≥ 0.15 g/mL, SBA ≥ 4 titer; men A, C: rSBA ≥8 titer or hSBA ≥4 titer) (Plotkin, 2010). On the other hand, the assay standardization has been successfully carried out only for Men A and C serogroups, using baby rabbit sera as a complement source. No standardization for Men B, W135, and Y has been established up to now. However, Borrow et al. evaluated and optimized parameters for interlaboratory reproducibility of the assay (Borrow et al., 2005). For MenB vaccine evaluation the hSBA titer ≥1:4 is considered the threshold for protective immunity against IMD (Borrow et al., 2020). No formal method has been established due to discrepant results between laboratories, which have been reconducted to the different epidemiology of prevalent strains, making the choice of a “universal” reference strain difficult. This is particularly true in the case of protein-based vaccines, where the antigens included in the vaccine formulation may not be expressed by all the different wild-type strains. For example, for MenB vaccines only strain-specific protein-based vaccines were developed to avoid the possibility of autoimmunity. As the protein antigens sequence and expression can vary among the same serotype, the vaccine coverage against circulating strains is difficult to establish (Donnelly et al., 2010). The bactericidal activity of the immunized serum samples, evaluated through SBA, increases with the number of antigens expressed by the strains and their affinity to vaccine antigens (Donnelly et al., 2010; Vogel et al., 2013). Consequently, the choice of the strains to be tested should consider this aspect for the immunogenicity assessment, especially to avoid very large and pointless efficacy trials (Donnelly et al., 2010). To evaluate the strain coverage of the multicomponent protein-based vaccine against MenB (4CMenB (Bexsero)) the Meningococcal Antigen Typing System (MATS) has been developed. The MATS is a sandwich ELISA that uses polyclonal rabbit antibodies raised against the three antigens included in the vaccine (factor-H-binding protein, neisserial heparin-binding antigen, and neisserial adhesin A) applied to bacterial lysates (Vogel et al., 2013). The authors find out that MATS relative potency could be used as a valid parameter to predict the killing in a large panel of strains (Donnelly et al., 2010; Vogel et al., 2013). For the same purpose, the MEASURE assay has been developed to predict the bactericidal response induced by the Bivalent rLP2086 (Trumenba) vaccine, composed of two variants of the factor H binding protein (fHbp) (McNeil et al., 2018). This assay, based on flow cytometry, can be used to evaluate the surface expression of a large collection of MenB isolates, using an anti-fHbp cross-reactive antibody. In this way, it becomes possible to predict the vaccine-induced strain susceptibility in SBA (McNeil et al., 2018). The complications in standardizing SBA for meningococcus Groups B, W135, and Y are the same as those encountered for other bacterial species. On the other hand, the evidence that vaccine-elicited functional antibodies can represent a valid serological CoP encouraged the development of SBA assay for multiple bacterial strains (Plotkin, 2010). The enumeration of the CFU as readout of the C-SBA influences several assay parameters, including the assay volume, the number of bacteria, the number of replicates, and the duration of the experiment. Consequently, the conventional SBA is considered labor-intensive, with a high inter-operator and inter-laboratories variability (Borrow et al., 2006). Even if the introduction of automated colony counting represented an improvement, the conventional assay still is not applicable to test a large number of serum samples (Necchi et al., 2017; Heuser et al., 2023). The need for an alternative high throughput SBA is derived from the awareness that antibody functionality is an important parameter to consider during vaccine efficacy assessment.

The Luminescence-SBA (L-SBA) applies to the same principles of the C-SBA but evaluates the antibody bactericidal activity by quantifying the ATP level as a correlate of bacterial survival (Figure 2B) (Necchi et al., 2017). In L-SBA the level of luminescence detected is directly proportional to the number of bacteria present in the assay wells, which is inversely proportional to the level of functional antibodies present in the serum (Mancini et al., 2022). Therefore, serum bactericidal titer obtained by the luminescence readout method strongly correlates with the data obtained by the conventional agar plate-based assay (Necchi et al., 2017). The L-SBA allows us to avoid the major disadvantages of the conventional SBA and can be used as a high throughput functional assay for vaccines’ immunogenicity evaluation. Shimanovich and co-authors have demonstrated that the SBA is a potential CoP for Shigella since it can evaluate the bactericidal activity elicited by vaccination, associated with a reduction of shigellosis in humans (Shimanovich et al., 2017). The equivalence of the results obtained with the L-SBA compared to the CFU-based method have been demonstrated for several pathogens, including *Citrobacter freundii*, *Salmonella serovars Typhimurium* and *Enteritidis*, *Shigella flexneri* serotypes *2a* and *3a*, *Shigella sonnei*, *Neisseria meningitidis* (Aruta et al., 2021). The same team has also demonstrated the applicability of a High-Throughput L-SBA for 9 bacterial species: *S. sonnei*, *S. flexneri 1b*, *S. flexneri 2a*, *S. flexneri 3a*, *S. typhimurium*, *S. enteritidis*, *S. paratyphi A,* and *C. freundii* along with a further optimization using 384-wells-plate format instead the 96-wells one (Aruta et al., 2021). Another version of the conventional SBA is represented by the use the Resazurin (R-SBA), a metabolic indicator that gives a fluorescence readout when is irreversibly reduced to resorufin by living bacteria. Stazzoni and co-authors developed this method for screening monoclonal antibodies against *Neisseria gonorrhea* and compared it to L-SBA and C-SBA (Stazzoni et al., 2023). The same fluorescent readout has been applied to develop a high-throughput version of SBA for meningococcal strains which maintains a good agreement with the conventional assay results (Mak et al., 2011).

### 5.3 The opsonophagocytosis assay

**Figure 3:**
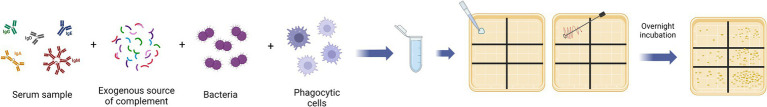
Graphical representation of the main steps for the opsonophagocytosis assays (OPA). The assay reaction mix is composed of complement, bacteria, phagocytic cells, and serially diluted serum samples from the immunized patients. After the incubation time, the reaction mix is plated and incubated overnight. The day after the colony counting of each plate is performed. The readout is the colony formation unit (CFU) value for each sera dilution point, which is proportional to the number of survival bacteria. This figure was created using BioRender.com.

The immune response elicited by vaccination involves different mechanisms of action. Depending on which bacterial infection occurs, functional antibodies mediate the clearance of the pathogens by complement activation and cellular phagocytosis. The activation of the complement classical pathway and MAC formation is evaluated by SBA assay, described in chapter 5.2. Cellular phagocytosis is mediated by the opsonization of the bacteria with antibodies and the C3b derived from complement activation (Feavers and Walker, 2010). The presence of opsonophagocytic activity is evaluated by the Opsonophagocytosis assay (OPA). Generally, OPA assay requires serially diluted sera incubated together with bacteria, complement, and phagocytic cells (Figure 3) (Boero et al., 2023). Depending on the phagocytic differentiation stage of the cells used in the assay, it is possible to evaluate the neutralizing activity or only the internalization of the bacteria. The use of non-differentiated cells in the assay, like HL-60 monocytes, indicates antibody-mediated uptake of bacteria. Flow cytometry-based OPA with non-differentiated cells can be used to evaluate the adherence/internalization using fluorescent whole bacteria or fluorescent beads coated with the antigens of interest (Boero et al., 2023). On the other hand, the most established OPA is the opsonophagocytic killing assay (OPKA) which requires the use of specialized cells, like differentiated HL-60 macrophages, and evaluate the killing efficiency (Boero et al., 2023). As for the SBA assay, the complement source needs to be previously tested for its intrinsic killing activity, in the absence of specific antibodies, and chosen based on the possible interference with the bacteria used in the assay (Feavers and Walker, 2010). Different cell lines can be used in the OPA, however, the HL-60 human leukemic cell line differentiated into activated neutrophils is the most frequently used and optimized as a phagocytic population because of their strong phagocytic activity. The conventional OPA assay evaluates the phagocytosis via CFU counting readout by plating the reaction mix in a solid agar plate after the incubation time. The IC_50_ is calculated by plotting the CFU count against serum concentration and it is considered a measure of antibody opsonizing capacity (Feavers and Walker, 2010). As for the SBA, the CFU-based method has several disadvantages: it is labor-intensive, time-consuming, and not suitable for the evaluation of a large number of samples (Necchi et al., 2017). The flow cytometry-based OPA can overcome some disadvantages related to plating and counting the CFU but cannot distinguish the adherence from the internalization of the bacteria (Fabbrini et al., 2012). Then, the development of multiplexed OPA (MOPA) allowed the test of up to four different serotypes in the same assay plate, taking advantage of the use of different antibiotics to select resistant strains (Kim et al., 2003). Furthermore, the automated colony counting tool allowed the acceleration of the process (Heuser et al., 2023). The MOPA is particularly useful in the case of a polyvalent Pneumococcal Conjugate Vaccine (PCV) clinical test, which would require performing an OPA assay against several serotypes (Romero-Steiner et al., 2006; Song et al., 2013). ELISA antibody levels at 0.35 mcg/ml remain an overall cut-off for the prevention of invasive pneumococcal disease (IPD) (Oosterhuis-Kafeja et al., 2007). For the OPA assay, the protective titer varied from 1/4 for type 1 to 1/769 for type 7F (Holmgren et al., 2017; Toh et al., 2021). In general, the OPA assay has been used to evaluate the presence of opsonizing antibodies for different bacterial vaccines, but it is currently recognized as a co-CoP only for PCV. The OPA has become an important method to evaluate the immunogenicity in PVC vaccine trials particularly for adults and the elderly, who may be susceptible to pneumococcal infections despite “adequate” or normal IgG antibody levels (LaFon and Nahm, 2018). For this reason, a standardized protocol for multiplexed opsonophagocytic killing assay for antibodies against *S. pneumoniae* is available and updated periodically (Nahm and Burton, 2014). A fluorescent version of the OPA (fOPA) based on flow cytometry analysis has been developed to evaluate the functional antibodies against Group B *Streptococcus.* The method takes advantage of the use of bacteria labeled with pHrodo™, which is a dye that emits bright fluorescence signals in the presence of acid conditions, like the phagolysosome compartments. In this way, the fluorescence signal is proportional to the level of the bacteria internalized by the HL-60 differentiated cells. The advantage over another type of flow cytometry-based OPA is the ability to measure only internalized bacteria excluding those that are only in adherence to the cells (Fabbrini et al., 2012). Concerning *Shigella* spp., no immunological correlates have been established and conventional OPKA assays have been used to evaluate the killing of opsonized bacteria with serum from vaccinated patients (Shimanovich et al., 2017; Mancini et al., 2023). The OPA assay has been also used to assess the opsonization ability of the antibody induced by a live attenuated vaccine against non-typhoidal *Salmonella* (Nasrin et al., 2021). The OPA has also been used to evaluate the immunogenicity of the novel 4-antigen *Staphylococcus aureus* vaccine (SA4Ag) and the 10-Valent Extraintestinal Pathogenic *Escherichia coli* Bioconjugate Vaccine (VAC52416) (Inoue et al., 2018; Fierro et al., 2023). For MenB vaccines, the OPA assay has been performed to assess protection in patients with primary terminal complement deficiency, whereas the complement-antibody mediated killing (SBA assay) cannot be assessed (Toh et al., 2021).

## 6 Discussion and conclusion

In recent years, the field of vaccinology has achieved significant advances. These innovations have indirectly led to the improvement of serological assays to assess the efficacy of vaccines and to better understand the mechanisms behind protective immunity. The immunogenicity is evaluated during all the steps performed during the approval process of a novel vaccine candidate, starting from the pre-clinical until the phase III clinical studies. The primary goal of a candidate vaccine is the prevention of the disease by inducing a persistent immunologic memory and the antibodies play a fundamental role. Antibodies prevent infection by enhancing different mechanisms, such as pathogens and toxin neutralization, complement activation, and cellular phagocytosis. Currently, the ELISA assay is the most widely used assay to measure the antibody concentration. However, the binding assay cannot evaluate the functionality of the antibodies. SBA and OPA are the most commonly used assays for the assessment of neutralizing and opsonophagocytic antibodies. However, all the conventional versions of these assays are labor-intensive, time-consuming, and not applicable to a large number of samples. For these reasons, high throughput versions of these assays have been proposed to overcome some of the drawbacks. Luminescence-SBA has been demonstrated as a good alternative to the CFU-based method for several pathogens, however, it is not yet considered the gold standard like conventional SBA for meningococcal strains. The availability of biological standards or pathogen-specific positive controls will be also critical to assess vaccine-induced immune responses, promote standardization, minimize inter and intra-laboratory variability, and support comparability between vaccine candidates. Despite numerous improvements, further optimizations and development are needed to reduce assay variability and increase the applicability. The final goal is the employment of functional assays as part of the vaccine immunogenicity assessment process for all bacterial species, especially considering the need for new vaccines against AMR bacteria that represent the future worldwide challenge.

## Author contributions

GF: Writing – original draft, Writing – review & editing. GB: Writing – review & editing. FV: Conceptualization, Writing – review & editing. ElM: Writing – review & editing. AM: Supervision, Writing – review & editing. EmM: Supervision, Writing – review & editing.
